# Recent Advances in the Synthesis of Spiroheterocycles via *N*-Heterocyclic Carbene Organocatalysis

**DOI:** 10.3390/molecules22111882

**Published:** 2017-11-08

**Authors:** Yue Liu, Xiang Zhang, Rong Zeng, Ying Zhang, Qing-Song Dai, Hai-Jun Leng, Xiao-Jun Gou, Jun-Long Li

**Affiliations:** 1 Antibiotics Research and Re-Evaluation Key Laboratory of Sichuan Province, Sichuan Industrial Institute of Antibiotics, Chengdu University, Chengdu 610052, China; lucindalau1225@sina.com (Y.L.); zhangxiang315@mails.ucas.ac.cn (X.Z.); m18428302851@163.com (R.Z.); cdudqs@163.com (Q.-S.D.); lnavy@126.com (H.-J.L.); gouxj@163.com (X.-J.G.); 2 Chengdu Institute of Organic Chemistry, Chinese Academy of Sciences, Chengdu 610041, China; 3 College of Ethnic Medicine, Chengdu University of Traditional Chinese Medicine, Chengdu 611137, China; zy511129@163.com; 4 University of Chinese Academy of Sciences, Beijing 100049, China

**Keywords:** spiroheterocycles, *N*-heterocyclic carbene organocatalysis, diastereoselective synthesis, enantioselective synthesis

## Abstract

Spiroheterocycles are regarded as a privileged framework because of their wide distribution in various natural products and synthetic molecules and promising bioactivities. This review focuses on the recent advances in the synthesis of spiroheterocycles by using the strategy of *N*-heterocyclic carbene (NHC) organocatalysis, and is organized based on the stereoselectivity and the reactive intermediates. According to the stereochemistry, this review was divided into two main parts, covering racemic and enantioselective versions. In each part, we firstly describe the synthetic transformations using nucleophilic Breslow intermediates, and then discuss the reactions that employ electrophilic acylazolium or radical cation intermediates. With those distinct catalytic activation modes of NHC organocatlysis, we expect this synthetic protocol will possibly produce new molecules with structural novelty and complexity, which may warrant further research in the field of drug discovery.

## 1. Introduction

**Figure 1:**
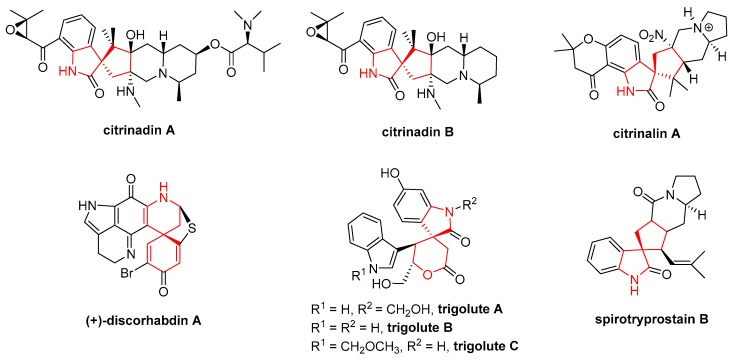
Select natural products containing spiroheterocycles.

Spiroheterocycles are a class of privileged scaffolds widely occurring in many natural products and pharmaceutical compounds [1,2,3]. Recently, spiroheterocycles have received special attention in medicinal chemistry because of their promising bioactivities, such as antitubercular, antiparasitic, and antitumor properties [2,4,5]. For example, citrinadins A and B isolated from *Penicillium citrinum* N059 strain show modest cytotoxicity against murine leukemia L1210 cells [6]. The alkaloid citrinalin A, a bioactive secondary metabolite of *Penicillium* species, exhibits antimycobacterial, cytotoxic and antifungal activities [7]. The marine alkaloid discorhabdin A inhibits protein-protein interactions, interrupting the interaction between hypoxia-inducible factor 1a (HIF-1a) and its transcriptional coactivator p300 [8]. Trigolutes A–C isolated from *Trigonostemon lutescens* twigs, are used in Thai folk medicine to cure food poisoning, asthma, and poisonous snake bites [9]. Spirotryprostatin B, which is a diketopiperazine alkaloid isolated from *Aspergillus fumigatus*, displays cell cycle inhibitory activity [10] (Figure 1). Due to their important physiological functions, the corresponding skeletons have therefore attracted great interest from synthetic chemists.

During the last two decades, with extensive and in-depth research on cycloadditions [11,12,13,14], spirocyclization reactions, such as alkylation [15,16], ring-expansion method [17], rearrangement- based approaches [18], the palladium-catalyzed [3+2] cycloaddition [19], [2+1] cycloadditions [20] and other methods, have been established to construct and modify these frameworks. Although significant advances have been achieved, constructing complex molecules with congested tetrasubstituted carbon stereocenters in one step under mild conditions still remains challenging [21,22,23,24].

**Figure 2:**
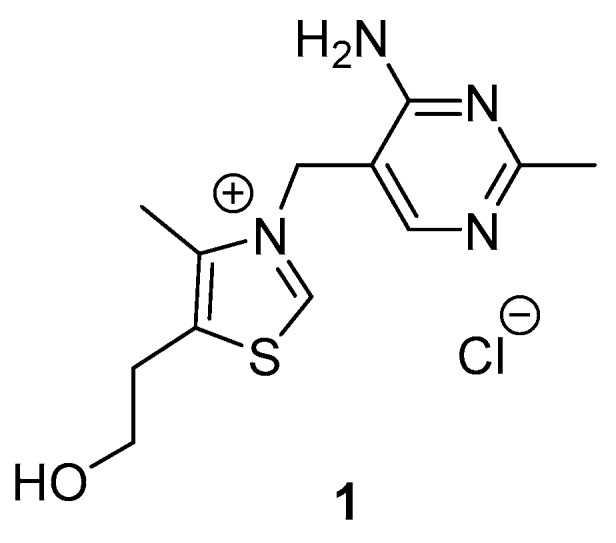
Coenzyme thiamine (vitamin B1).

In biological systems, many complicated biochemical transformations are catalyzed by enzymes; for example, the nucleophilic acylation reaction is catalyzed by transketolase enzymes [25], in the presence of a coenzyme named thiamine (**1**, Figure 2) [26]. This biocatalytic transformation is interesting to many chemists because it can help elucidate the nature and design of biomimic catalysis [27].

**Figure 3:**
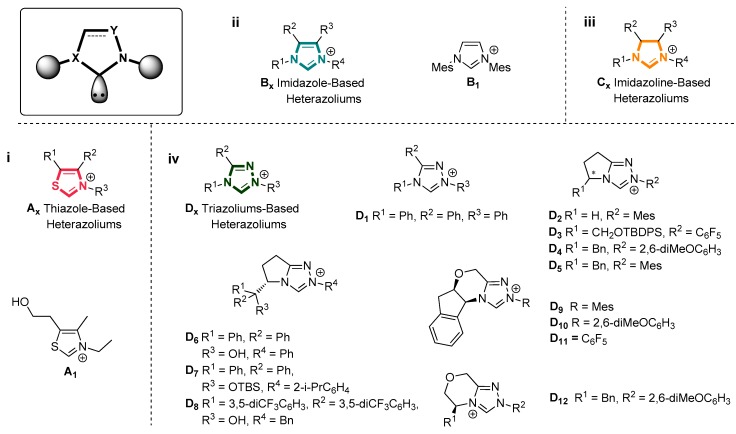
General types of *N*-heterocyclic carbenes.

Early carbene chemistry was developed around the 1900s [27,28,29,30,31,32]. Since Bertrand [33,34], Arduengo [35] and their colleagues reported stable nucleophilic carbenes, chemists have set off a gold rush in this field, and NHCs have been successfully applied to organocatalysis, thereby enabling various unconventional chemical transformations via diverse reactive intermediates [36,37,38,39]. According to their different core structures, NHCs can be divided into four types, namely, thiazolium, triazolium, imidazolium, and imidazolin-2-ylidenes [37]. Rovis et al., further summarized diverse NHC catalysts with different substituents (for details, see Figure 3) [40].

**Scheme 1:**
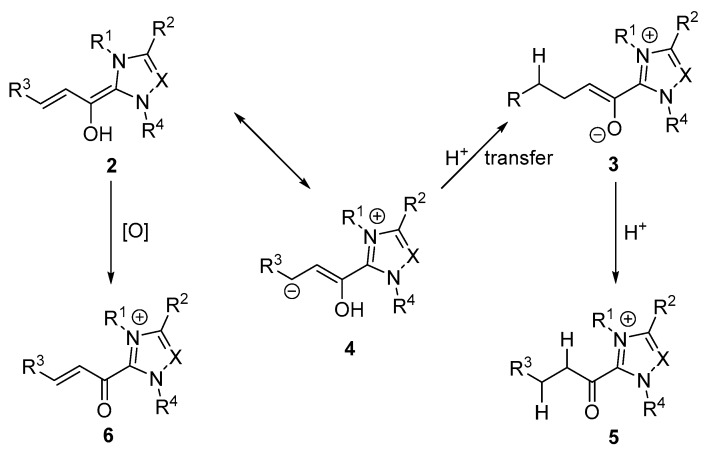
Transformation of related intermediates under certain conditions.

In the presence of these NHC catalysts, various reactive species can be generated from aldehydes and other carbonyls. For example, Breslow intermediate **2**, enolate **3**, homoenolate **4**, acylazolium **5**, and α,β-unsaturated acylazolium **6** are the most investigated. Among these species, Breslow intermediate, enolate, and homoenolate are typically used as nucleophiles, and acylazolium and α,β-unsaturated acylazolium are used as electrophilic species. Mutual transformation among these intermediates enables the unique NHC-catalyzed reactions (Scheme 1) [41].

This review includes recent examples of different catalysts and reactions used to synthesize spiroheterocycles via NHC organocatalysis. Examples have been selected to emphasize notable spirocyclization strategies and compare the reactivity and selectivity of different types of NHC organocatalysts. This synthetic protocol produces new spirocyclic molecules with structural novelty and complexity, which may warrant further research in the field of drug discovery.

## 2. NHC-Catalyzed Synthesis of Spiroheterocycles

### 2.1. Synthesis of Racemic Spiroheterocycles via NHC-Catalyzed Reactions

#### 2.1.1. Catalysis Involving Nucleophilic Breslow Intermediates

**Scheme 2:**
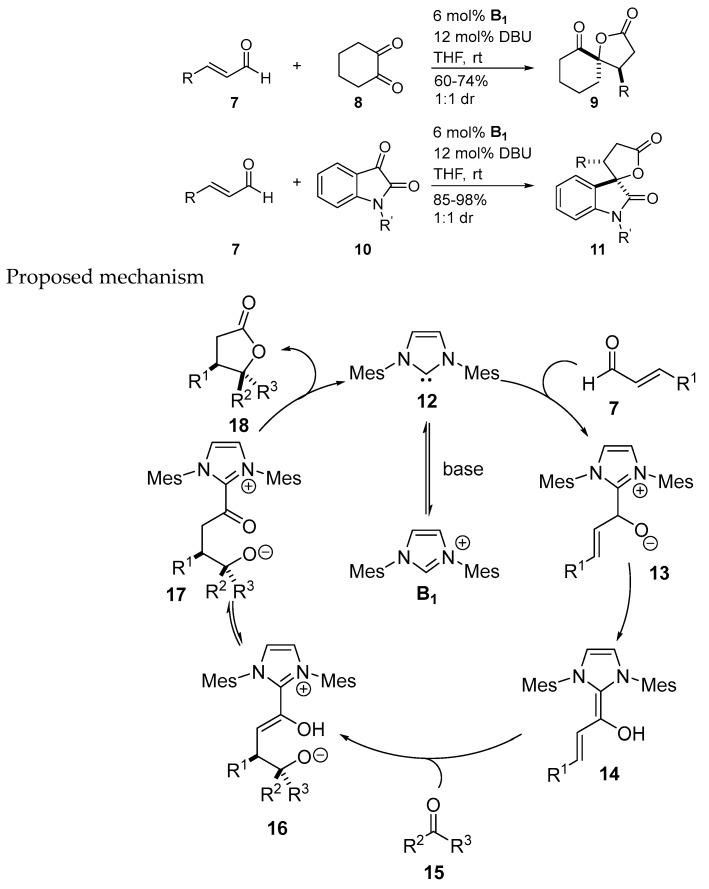
Synthesis of spirolactone-γ-lactone derivatives via **B_1_**-catalyzed reactions.

In 2006, Nair et al., reported the addition of enals to 1,2-dicarbonyl compounds via a homoenolate pathway, thereby opening a route to γ-spirolactones [42] (Scheme 2). In this reaction, α,β-unsaturated aldehydes **7** are used as nucleophiles in the presence of NHC catalyst, and 1,2-cyclohexanedione (**8**) and isatins **10** are used as competent electrophiles. The yield of spirocyclohexanone products **9** ranges from 60% to 74%, whereas spirooxindole γ-lactones **11** are obtained in 85–98% yields. However, the diastereoselectivity of this method (1:1 dr) was unsatisfactory. After addition of the NHC catalyst **12** to the aldehydes **7** and subsequent proton transfer in the intermediate **13**, this reaction begins with the nucleophilic addition of Breslow intermediates **14** to **15**, thereby giving enol azolium **16**. Tautomerization of **16** produces acylazolium **17**, which subsequently cyclizes to deliver the γ-lactone product **18** and release the catalyst.

**Scheme 3:**
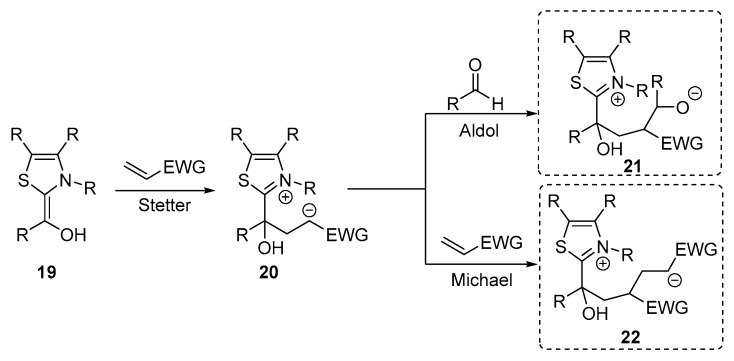
General Stetter–aldol or Stetter–Michael sequential reactions.

**Scheme 4:**
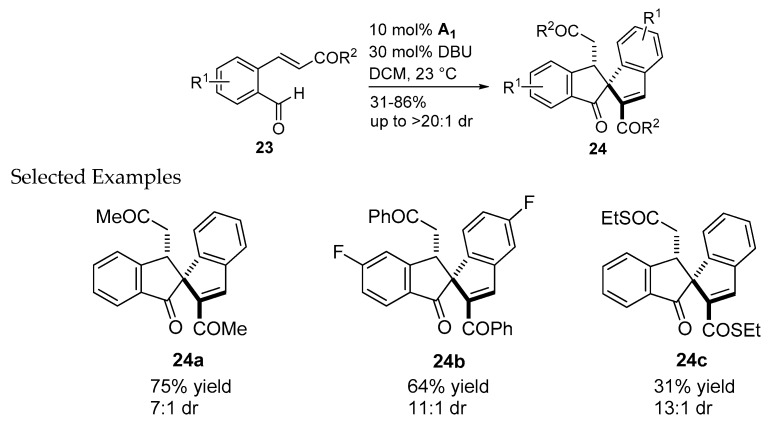
Synthesis of spiroindanes **24** via Stetter–aldol–Michael reaction.

Later, Gravel and co-workers developed one-pot intermolecular Stetter reactions coupled with intramolecular Michael or aldol reactions. In these reactions, after conjugate addition of the Breslow intermediate **19** to the Michael acceptor, enolate **20** is trapped by an aldehyde or an Michael acceptor and gives intermediate **21** or **22** (Scheme 3). Later on, they expanded this reaction to spiroindane skeletons. The corresponding spiroindanes **24** are synthesized from *o*-formylchalcone derivatives **23** in modest to good yields (31–86%) and with excellent diastereoselectivities (up to >20:1 dr) (Scheme 4) [43].

**Scheme 5:**
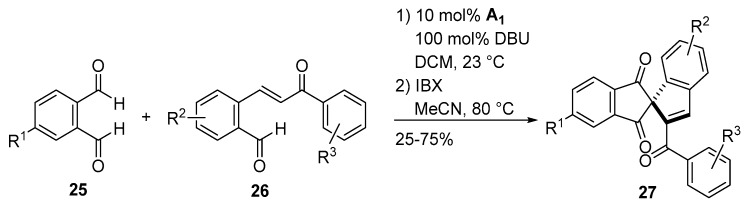

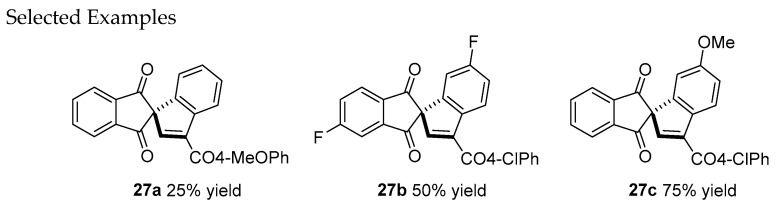
Synthesis of spiroindanes **27** via Stetter–aldol–aldol reaction.

In 2011, Gravel and co-workers exploited a Stetter–aldol–aldol reaction starting with phthaldialdehydes **25** and the acceptors **26** to generate spiro bis-indanes **27** in a diastereoselective manner (Scheme 5). Electron-donating groups (EDGs) considerably affect the reactivity of the reaction, resulting in a modest yield (25%); however, electron-withdrawing groups (EWGs) enhance the electrophilicity of the Michael acceptor, thereby reducing the reaction time and increasing the product yield (75%) [44].

**Scheme 6:**
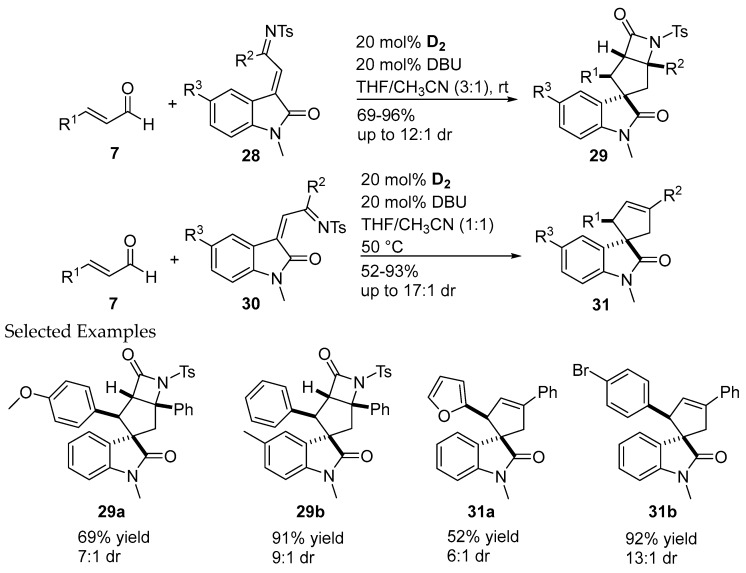
Synthesis of spirocyclic oxindoles **29** and **31** via **D_2_**-catalyzed cyclization.

Chi and colleagues developed a diastereoselective method for a facile access to spirocyclic oxindoles **29** and **31** containing two quaternary carbons. Enal-derived homoenolate intermediates with three consecutive reactive positions are used in the reaction, and a special oxindole-derived α,β-unsaturated imines with β,β-disubstituents **28** and **30** are the reaction partner. Initial studies have shown that catalysts based on an imidazolium skeleton are ineffective in this reaction, whereas the triazolium-based catalyst **D_2_** has proven to be promising. With this catalyst, the desired spiroindole products can be obtained in good to excellent isolated yields (69–96%) with moderate to good diastereoselectivity (2:1 to 12:1 dr) (Scheme 6) [45].

**Scheme 7:**
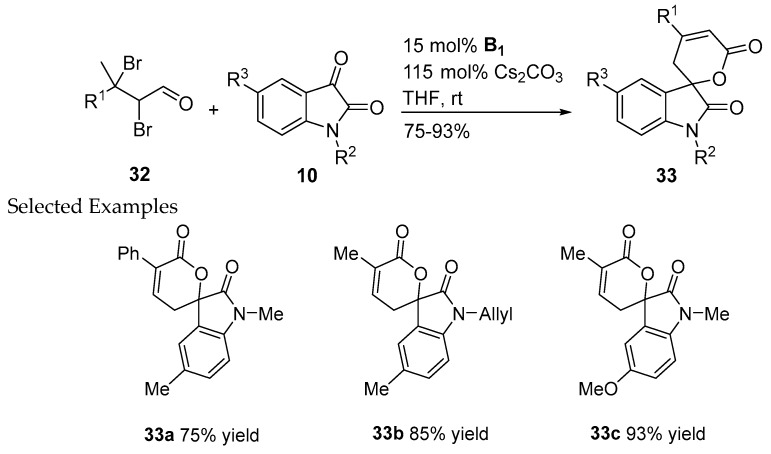
Synthesis of spirocyclic oxindole–dihydropyranones **33**.

**Scheme 8:**
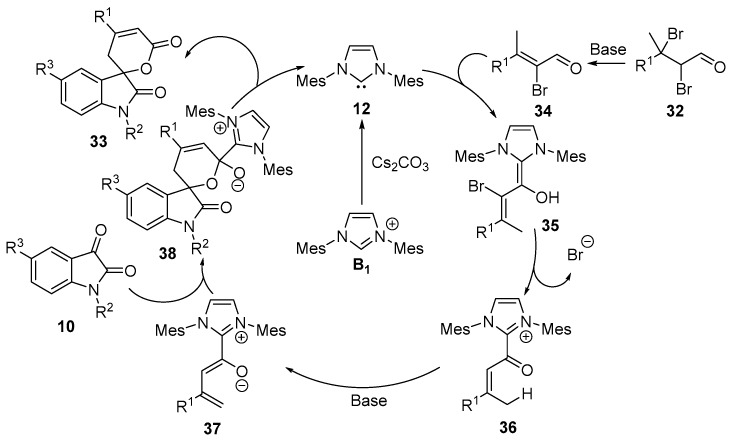
Proposed mechanism of the formation of spirocycles **33**.

In 2013, Yao and co-workers employed an efficient NHC-catalyzed [4+2] annulation of α,β-dibromoaldehyde **32** or α-bromo-α,β-unsaturated aldehydes **34** bearing γ-H with isatin derivatives to prepare spirocyclic oxindole–dihydropyranones **33** (Scheme 7). In this reaction, the condensation of the NHC catalyst **B_1_** and **34** produces Breslow intermediate **35**, which is subsequently oxidized to **36** through intramolecular debromination. Then, acylazoliumion **36** is deprotonated at the γ-position to give the vinyl enolate **37** under basic conditions. Afterward, intermediate **37** reacts with isatins **10** probably through an *oxo*-Diels–Alder reaction mechanism or a non-concerted nucleophilic addition followed by intramolecular cyclization for the target product **33** and and the catalyst is released from intermediate **38** (Scheme 8). The yield of spirocyclic oxindole–dihydropyranones in this reaction ranges from 75% to 93% [46].

**Scheme 9:**
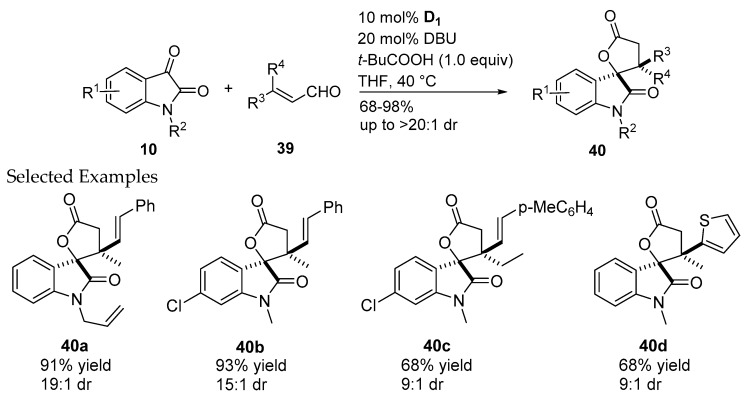
Synthesis of **40** via diastereoselective [3+2] annulations.

Later, Glorius and colleagues employed a conjugate umpolung strategy to synthesize spirooxindole scaffolds with contiguous quaternary stereocenters. The mechanism of this reaction involves an NHC catalyst and an enal **39** that initially form a tetrahedral intermediate and subsequently transform to the Breslow intermediate. In the presence of an acid co-catalyst, the species can react with isatin **10** via one of two possible pre-transition-state assemblies. The preference for the favored pathway leads to the observed major diastereoisomer. After an adduct diastereoselectively forms, an intramolecular alkoxide attack at the carbonyl group produces the desired spirooxindoles **40** and regenerates the NHC catalyst. The yields in this transformation range from 68% to 98%, and the diastereoselectivity is generally good (8:1 to >20:1 dr) (Scheme 9) [47].

#### 2.1.2. Catalysis Involving Acylazolium Intermediates

**Scheme 10:**
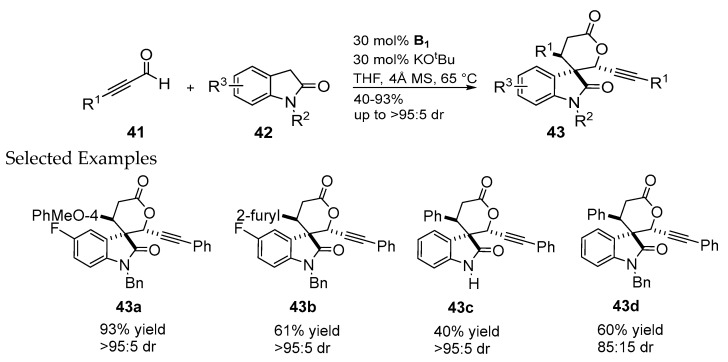
Synthesis of spirooxindole 4*H*-pyran-2-ones **43** from ynals.

In recent years, efforts have been devoted to investigating NHC-based acylazolium and azolium enolate intermediates. The most commonly used method to access NHC-derived α,β-unsaturated acylazolium intermediates is based on an internal redox activation of α-oxidizable aldehydes. Du, Lu and co-workers reported an effective strategy to synthesize spirooxindole 4*H*-pyran-2-one **43** derivatives through the NHC-catalyzed three-component domino reaction of oxindoles **42** and ynals **41**. This reaction provides moderate to good yields (40–93%) with excellent diastereoselectivity (up to >95:5 dr) (Scheme 10) [48].

**Scheme 11:**
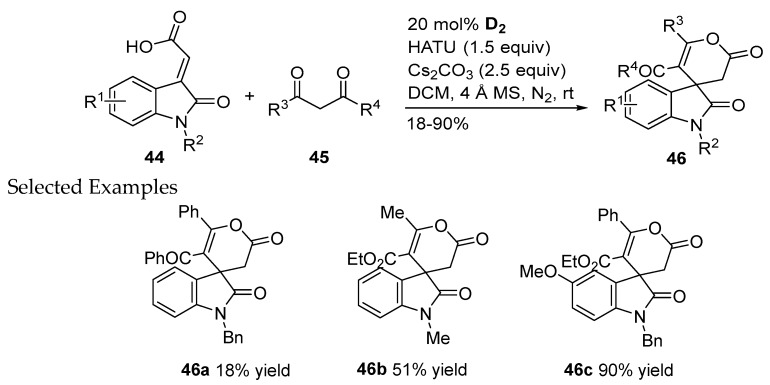
**D_2_**-catalyzed synthesis of spirooxindole lactones **46**.

In 2017, the same group published an NHC-catalyzed formal [3+3] annulation of isatin-derived α,β-unsaturated acids **44** with 1,3-dicarbonyl compounds **45** to synthesize 3,4′-spirooxindole lactones **46**. Of note, acid substrates are generally more bench stable than aldehydes; in this case, these substrates can be activated in situ (Scheme 11). Under optimized conditions, a wide range of substrates produce the corresponding products in moderate yields ranging from 18% to 90% [49].

**Scheme 12:**
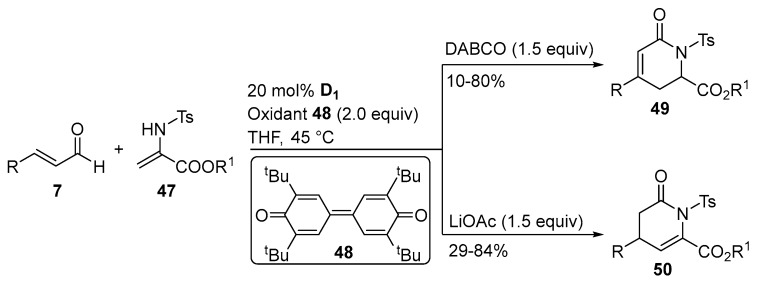
**D_1_**-catalyzed synthesis of dihydropyridinones **49** and **50**.

**Scheme 13:**
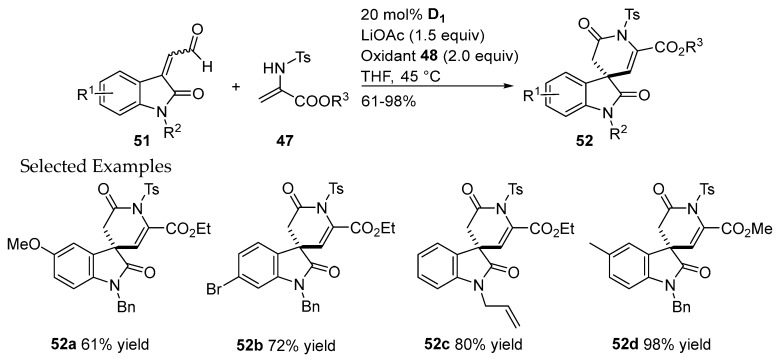
**D_1_**-catalyzed synthesis of spirooxindoles **52**.

Qi and co-workers established an NHC-catalyzed synthesis of dihydropyridinones and spirooxindoles. With the external oxidant **48**, the reaction proceeds via a [3+3] annulation of cinnamaldehydes **7** or isatin-derived enals **51** in the presence of 2-aminoacrylates **47** (Scheme 12). Two different dihydropyridinones **49** and **50** are generated through this novel strategy by using different bases, and a series of spirooxindole derivatives **52** is also synthesized in moderate to good yields (61–98%) (Scheme 13) [50]. 

### 2.2. Synthesis of Chiral Spiroheterocycles via NHC-Catalyzed Reactions

#### 2.2.1. Catalysis Involving Chiral Nucleophilic Breslow Intermediates

**Scheme 14:**
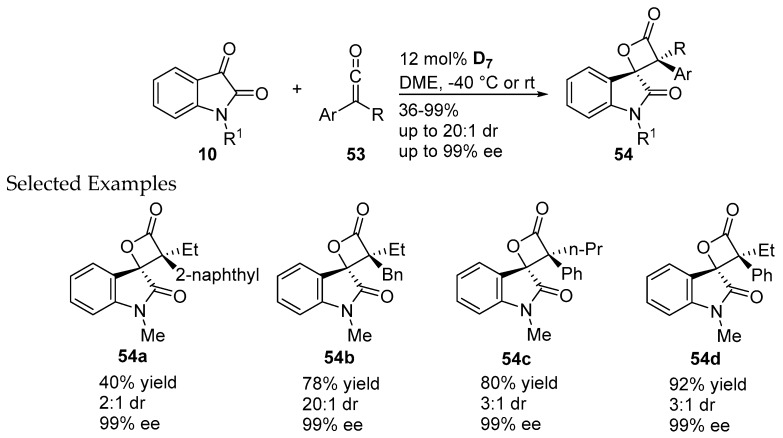
Enantioselective [2+2] cycloaddition of isatins **10** and ketenes **53**.

Ye and colleagues demonstrated an enantioselective NHC-catalyzed cycloaddition reaction that generates spirocyclic oxindole-β-lactones **54** from ketenes **53** and isatins **10** through a formal [2+2] annulation (Scheme 14). This reaction efficiently forms products in good yields (36–99%) with good diastereoselectivities (3:1 to >20:1 dr) and high enantioselectivities (83–99% ee) [51].

**Scheme 15:**
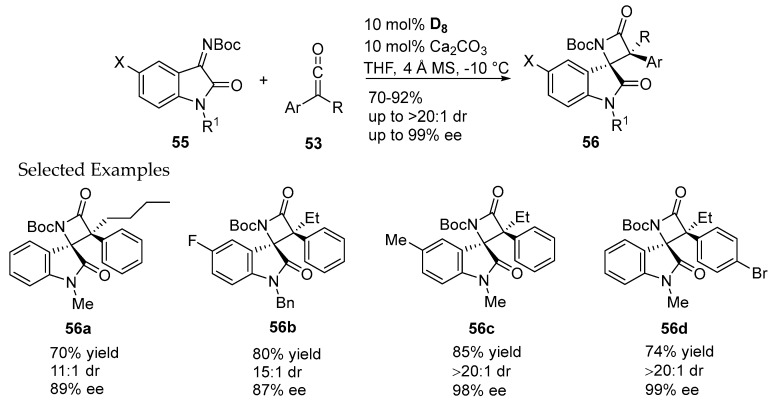
Enantioselective Staudinger reaction of ketenes **53** and isatin-derived ketimines **55**.

Ye and co-workers further demonstrated that bifunctional NHC with a free hydroxyl group is an efficient catalyst for the enantioselective Staudinger reaction of ketenes **53** with isatin-derived ketimines **55** (Scheme 15), thereby producing the corresponding spirocyclic oxindolo-β-lactams **56** in high yields (70–92%) with excellent diastereoselectivities (up to >20:1 dr) and enantioselectivities (87–99% ee) [52].

**Scheme 16:**
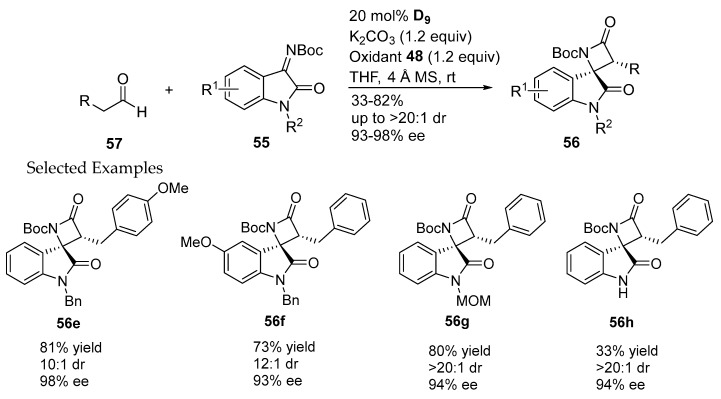
Synthesis of spirooxindole β-lactams **56** via NHC-catalyzed oxidative [2+2] annulation.

Xu and Ren developed an NHC-catalyzed oxidative [2+2] annulation reaction to generate spiro-β-lactams **56** bearing two vicinal stereogenic centers from simple aliphatic aldehydes **57** and isatin-derived ketimines **55**. This reaction efficiently delivers products in 33–82% yield, 93–98% ee, and 5:1 to 20:1 dr. The remarkable features of this formal [2+2] annulation reaction include direct carbon functionalization of aliphatic aldehydes, easily accessible starting materials, mild reaction conditions, and readily removable protecting groups (Scheme 16) [53].

**Scheme 17:**
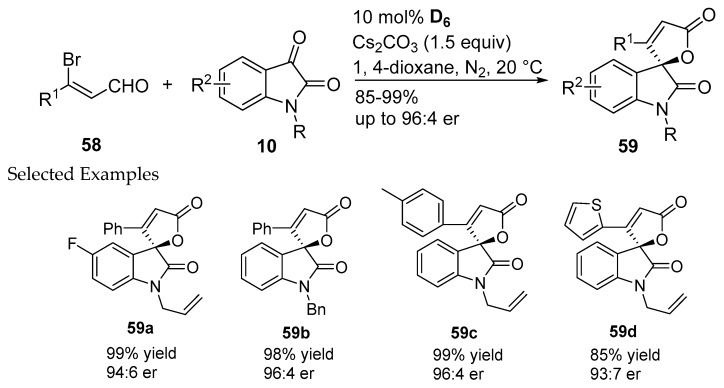
Asymmetric synthesis of the spirooxindole **58** via [3+2] annulation.

In 2014, Ma group illustrated an asymmetric formal [3+2] annulation of aryl 3-bromoenals **58** and isatins **10** to produce spirooxindole–butenolides **59** in excellent yield with high enantioselectivity through hydrogen-bonding activation-assisted chiral NHC catalysis (Scheme 17). This reaction provides excellent yield (85–99%) and high enantioselectivity (93:7–96:4 er) [54].

**Scheme 18:**
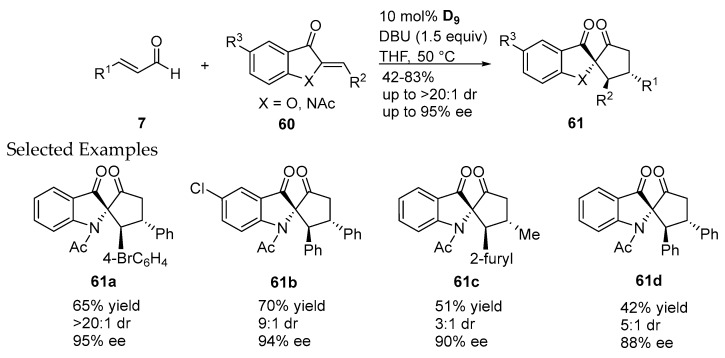
NHC-catalyzed [3+2] annulation of α,β-unsaturated aldehydes **7** and aurones **60**.

In the same year, Glorius and co-workers developed a highly enantioselective NHC-catalyzed formal [3+2] annulation of α,β-unsaturated aldehydes **7** with azaaurones or aurone **60**. This reaction begins with an initial conjugate addition of homoenolate to a Michael acceptor. The resultant enol−azolium tautomerizes to acylazolium. After the pendant undergoes cyclization, the enolate liberates free carbenes and affords the desired spiro-products **61**. This transformation provides moderate to good yields ranging from 42% to 83%, a generally good diastereoselectivity (3:1 to >20:1 dr) and an excellent enantioselectivity (88–95% ee) (Scheme 18) [36].

**Scheme 19:**
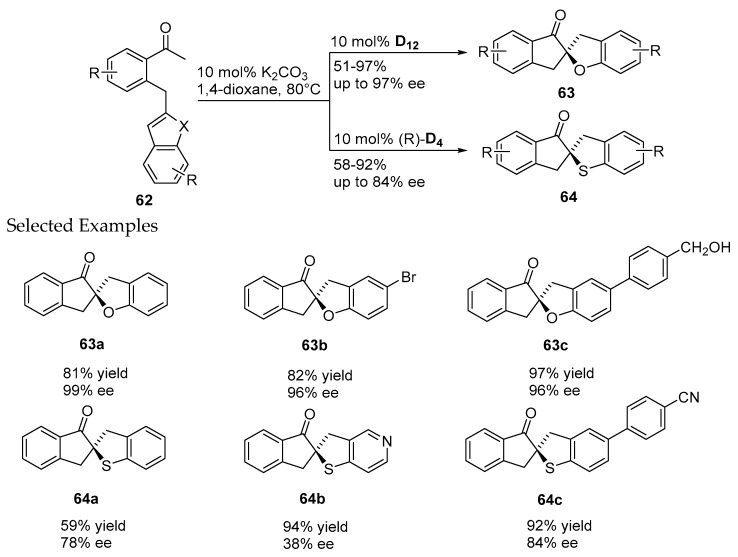
NHC-catalyzed enantioselective hydroacylation of benzoheterocycle **62**.

In 2016, Glorius and colleagues successfully used modified benzofurans and benzothiophenes **62** as substrates in an enantioselective NHC-catalyzed intramolecular hydroacylation/dearomatization transformation (Scheme 19). This reaction provides access to a class of scarcely explored spirocycles **63** and **64** with up to 99% ee in modest to good yields (51–97%). The products bear interesting three-dimensional pseudo-axial chirality and shows a typical ketone reactivity [55].

**Scheme 20:**
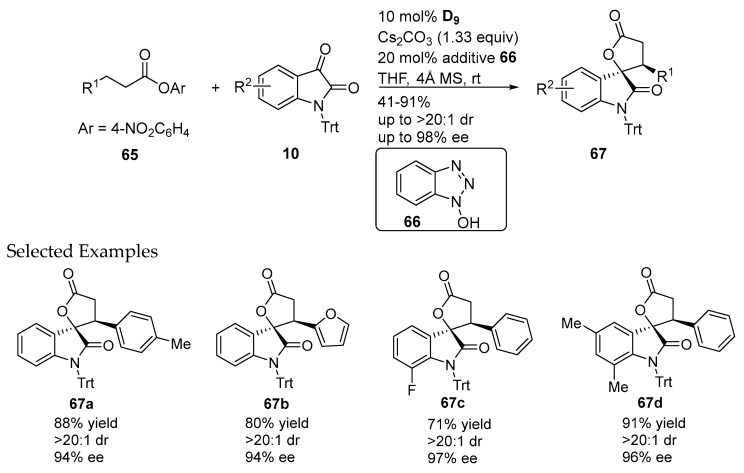
Asymmetric synthesis of spirooxindole lactones **67**.

Scheidt’s group constructed chiral spirooxindole lactones **67** using a Lewis acid in conjunction with an NHC without additives [56,57]. After Scheidt’s work, Xu et al., described a 1-hydroxy- benzotriazole (HOBt, **66**)-assisted, NHC-catalyzed direct β-functionalization reaction of saturated carboxylic esters **65** that undergo a formal [3+2] annulation with isatins **10** in a highly efficient, diastereoselective, and enantioselective manner to afford chiral spirooxindole lactones **67**. Notably, the use of a catalytic amount of HOBt in this reaction remarkably improves diastereoselectivity (>20:1 dr) and enantioselectivity (58–98% ee) (Scheme 20) [58].

**Scheme 21:**
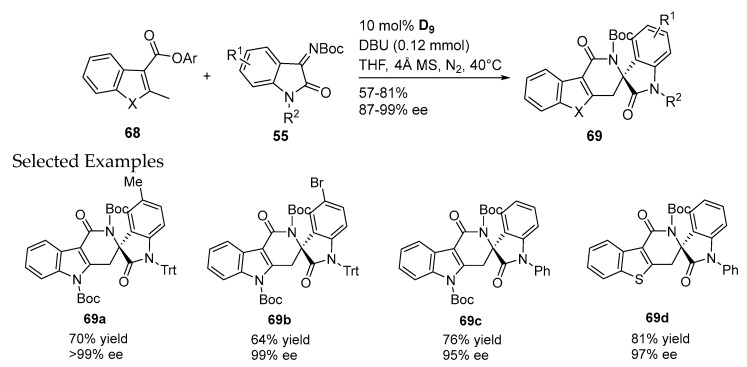
**D_9_**-Catalyzed [4+2] annulation reactions for the synthesis of **69**.

Later on, Xu and co-workers developed an efficient reaction to produce heterocyclic *ortho*-quinodimethanes **69** from 2-methyl-heteroarene-3-carboxylic esters **68** through NHC catalysis. The heterocyclic *ortho*-quinodimethanes generated in situ behave as 1,4-dipolarophiles to undergo a formal [4+2] annulation reaction with isatin-derived ketimines. The reaction affords chiral heteroarene-fused δ-lactams bearing a quaternary stereogenic center in moderate to good yields ranging from 57% to 81% and with high to excellent enantioselectivities (87 to >99% ee) (Scheme 21) [59].

**Scheme 22:**
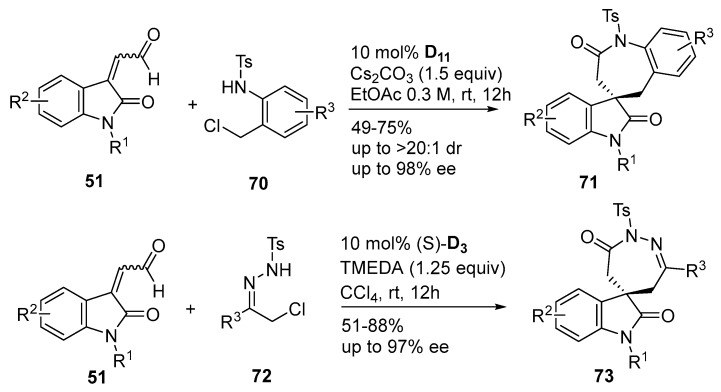
Asymmetric synthesis of spirobenzazepinones **71** and spiro-1,2-diazepinones **73**.

Very recently, Enders and co-workers reported an NHC-catalyzed asymmetric synthesis of spirobenzazepinones **71**, spiro-1,2-diazepinones **73** via [3+4]-cycloaddition reactions of enals derived from isatins **51** and aza-*o*-quinone methides or nitrosoalkenes in situ from *N*-(*o*-chloro-methyl)aryl amide **70** or a-halohydrazones **72**. The spirocyclic products containing a seven-membered ring are synthesized in good yields with excellent enantioselectivities (Scheme 22) [60].

**Scheme 23:**
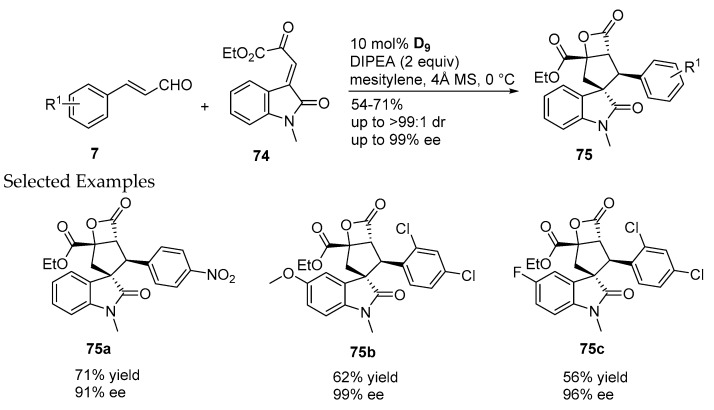
Asymmetric synthesis of β-propiolactone-fused spiro[cyclopentane-oxindoles] **75**.

Wang and co-workers developed a cascade asymmetric Michael-intramolecular aldol-lactonization of enals **7** with oxindolyl-β,γ-unsaturated α-ketoesters **74**. In this reaction, the desired β-propiolactone-fused spiro[cyclopentane-oxindoles] **75** contain four contiguous stereocenters, including a spiro all-carbon center and a quaternary carbon center. A variety of chiral NHC catalysts and bases have been investigated, and the results have shown that NHC **D_9_** and DIPEA can provide the highest enantioselectivity (up to 99% ee) and diastereoselectivity (>99:1 dr) (Scheme 23) [22].

#### 2.2.2. Catalysis Involving Chiral Acylazolium Intermediates

**Scheme 24:**
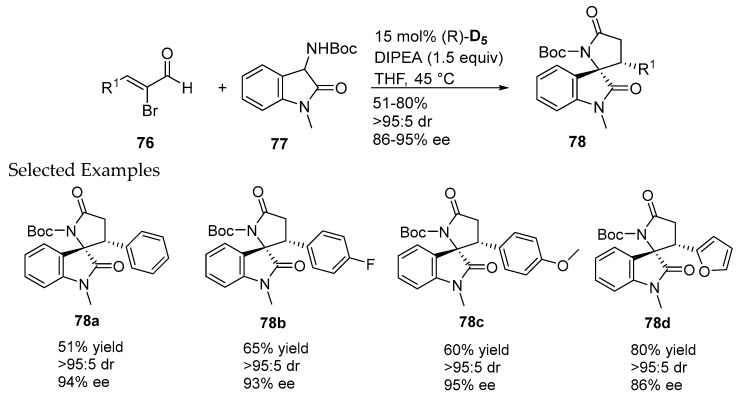
Asymmetric synthesis of spirooxindole γ-butyrolactams **78**.

In recent years, many research groups have made impressive contributions to the applications of α,β-unsaturated acyl azoliums generated from different precursors, such as ynals, enals, α,β-unsaturated acyl fluorides, and esters. After Yao’s work [61], Du and colleagues reported a formal [3+2] annulation of α-bromoenals **76**-derived α,β-unsaturated acyl azoliums with 3-amino-oxindoles **77** via NHC organocatalysis. The functionalized spirooxindole γ-butyrolactams **78** are synthesized with high diastereoselectivities (up to >95:5 dr) and enantioselectivities (86–95% ee) (Scheme 24) [62].

**Scheme 25:**
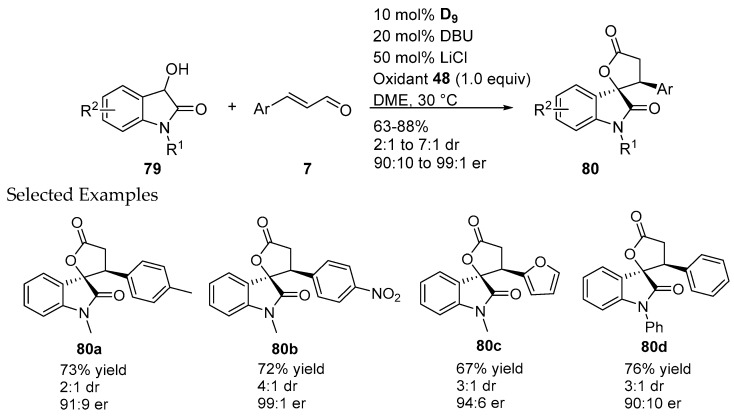
Synthesis of chiral spiro γ-butyrolactones **80** via [3+2] cyclization.

Biju, Yetra and co-workers presented an enantioselective NHC-catalyzed annulation of enals **7** with 3-hydroxy oxindoles **79**, resulting in the formation of spiro γ-butyrolactones **80**. The products are formed in moderate to good yields (63–88%) and with good enantioselectivity (up to 99:1 er) and diastereoselectivity (up to 7:1 dr). The reaction likely proceeds via the generation of the chiral α,β-unsaturated acyl azolium intermediate, followed by its interception with oxindoles in a formal [3+2] cyclization to afford the spiro compounds (Scheme 25) [63].

Recently, Qi and colleagues reported an efficient strategy to access 5,6-dihydropyridinones, 3,4-dihydropyridinones and spirooxindoles via the NHC-catalyzed [3+3] annulation of 2-aminoacrylates with cinnamaldehydes and oxindole-derived enals. Moreover, two different dihydropyridinones were produced by using this novel strategy with two different bases, namely, DABCO and LiOAc. They also synthesized a series of spirooxindole products in moderate to good yields (61–98%). An asymmetric catalytic version of this methodology has been conducted to investigate this novel strategy, and the desired product yields range from 67% to 83% with up to 99% ee [50].

**Scheme 26:**
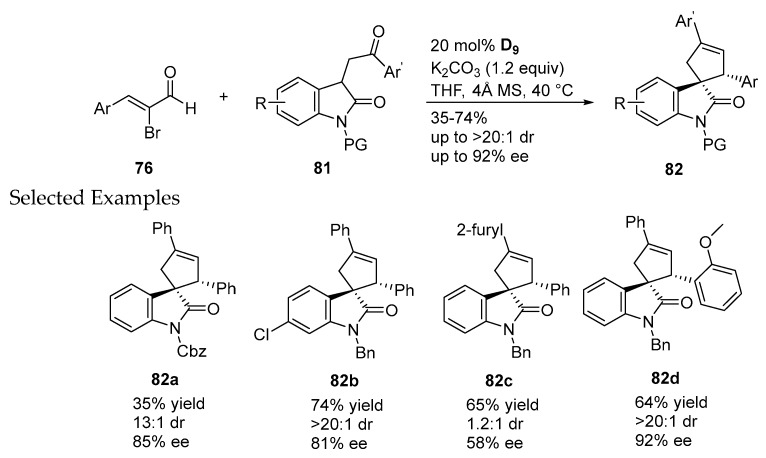
Asymmetric synthesis of spirocyclopentene-2-oxindoles **82.**

In 2017, Ye and co-workers developed an NHC-promoted synthesis of chiral spirocyclopentene-2-oxindoles **82** via a Michael–aldol–lactonization decarboxylation cascade of bromoenals **76** and oxindoles **81**. The spirocyclopentene-2-oxindoles bearing two contiguous stereocenters are obtained in good yields (35–74%) with good to excellent diastereoselectivity (up to >20:1 dr) and high enantioselectivities (up to 92% ee) (Scheme 26) [64].

**Scheme 27:**
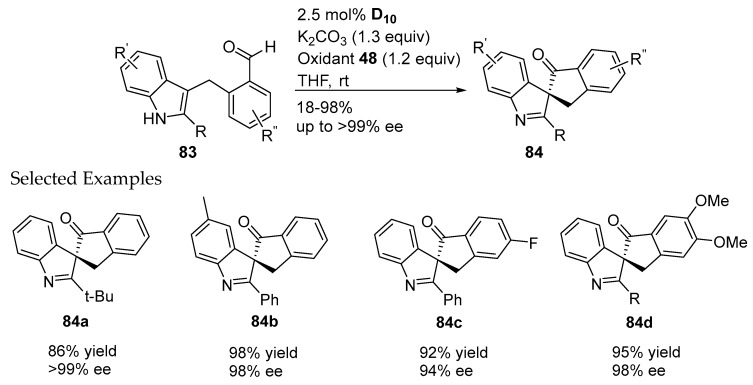
Oxidative NHC-catalyzed dearomatization to prepare spirocyclic indolenines **84**.

An asymmetric intramolecular dearomatization of indoles using oxidative NHC catalysis was reported by Studer and co-workers. In this reaction, the NH-free indolyls **83** as starting materials, can be easily transformed to valuable spirocyclic indolenines **84** with an all-carbon quaternary stereocenter through catalytic asymmetric dearomatization of an indole core (Scheme 27). The products form in good yields of up to 98% and >99% ee are obtained [65].

#### 2.2.3. Catalysis Involving Radical Cation Intermediates

**Scheme 28:**
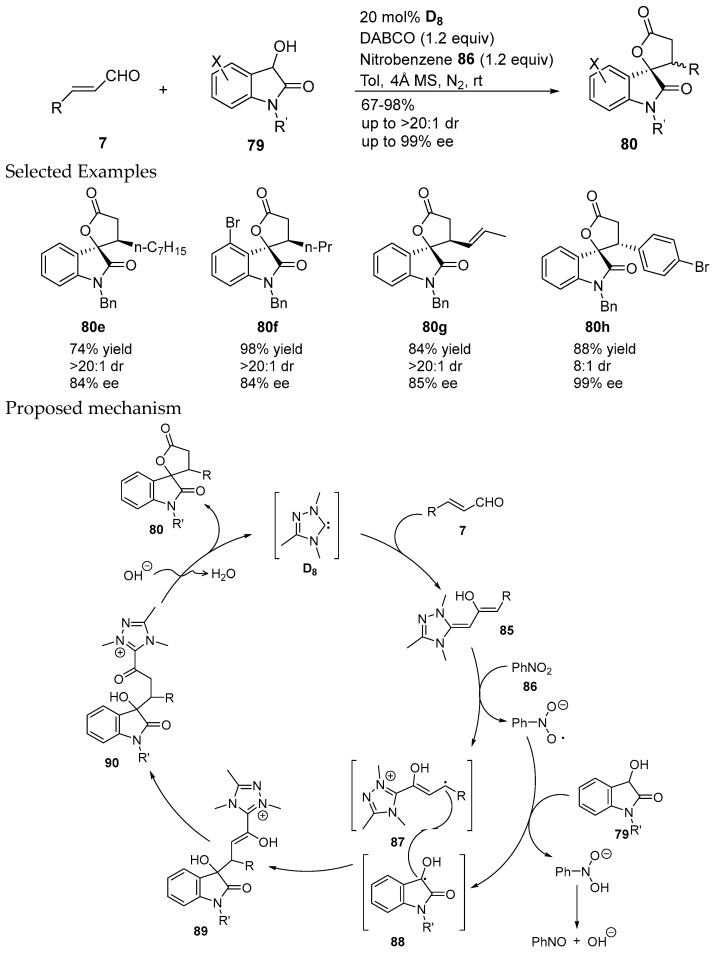
Enantioselective oxidative [3+2] annulation of enals.

In addition to the aforementioned synthesis of spiroheterocycles via Breslow and acylazolium intermediates, the oxidative cross-coupling of homoenolate and enolate remains unexplored. Ye, Sun and co-workers developed an NHC-catalyzed oxidative cross-coupling of dioxindoles **79** and enals **7**. The corresponding spirooxindole-γ-lactones **80** are generated in good yields (67–98%) with high to excellent diastereoselectivities (6:1 to >20:1 dr) and enantioselectivities (80–99% ee). This NHC-catalyzed oxidative [3+2] annulation reaction of dioxindoles and enals is proposed to occur via a radical-radical cross-coupling pathway (Scheme 28). 

## 3. Conclusions

Firstly, Breslow intermediate **85** is formed by the addition of **D_8_** and enals **7**. Subsequently, in the presence of nitrobenzene **86**, Breslow intermediate is oxidized to the radical cation intermediate **87**. Meanwhile, the cross-coupling of the homoenolate radical **87** and enolate radical **88** generated from dioxindole **79** affords adduct **89**, which is tautomerized to acylazolium **90**. Finally, with the assistant of base, the lactonization of **90** generates the spiro-product **80** and releases the NHC catalyst [66]. In the past decades, spiroheterocycles have attracted great interests among the scientific community due to their special physiological and pharmacological properties, such as antitubercular, antiparasitic, antifungal and antitumor activities [8,67,68]. Thus, chemists have made a lot of efforts on the synthesis of the corresponding skeletons to find promising compounds that might be useful in the area of drug research and development.

This review highlights the recent application of NHC organocatalysis in the synthesis of spiroheterocyles, and the aforementioned excellent works show that complex molecular skeletons can be constructed efficiently and rapidly by using simple starting materials under mild conditions. Although certain NHC catalysts and strategies are possible “privileged” routes for the efficient construction of spirocyclic derivatives, several problems, such as high catalyst loading and sensitivity to water and air, remain unsolved and thus need further investigations to develop new strategies. Nevertheless, we believe that the existing synthetic protocols and the developing transformations will provide powerful and efficient NHC organocatalytic reactions for the continuous construction of useful spiroheterocycles.
